# Supplementary material for: Molecular genetic aetiology of general cognitive function is enriched in evolutionarily conserved regions
Source: Transl Psychiatry. 2016 Dec 13;6(12):e980–. doi: 10.1038/tp.2016.246 (PMC5290340; doi:10.1038/tp.2016.246)
Supplement: Supplementary Figures and Table 1 [file tp2016246x1.docx]

Supplementary Figure 1. Enrichment analysis for general cognitive function using the 52 functional categories in the baseline model. The enrichment statistic is the proportion of heritability found in each functional group divided by the proportion of SNPs in each group (Pr(h^2^)/Pr(SNPs)). The dashed line indicates no enrichment found when Pr(h^2^)/Pr(SNPs) = 1. Statistical significance is indicated by asterisk. FDR correction indicates significance at 0.0095.


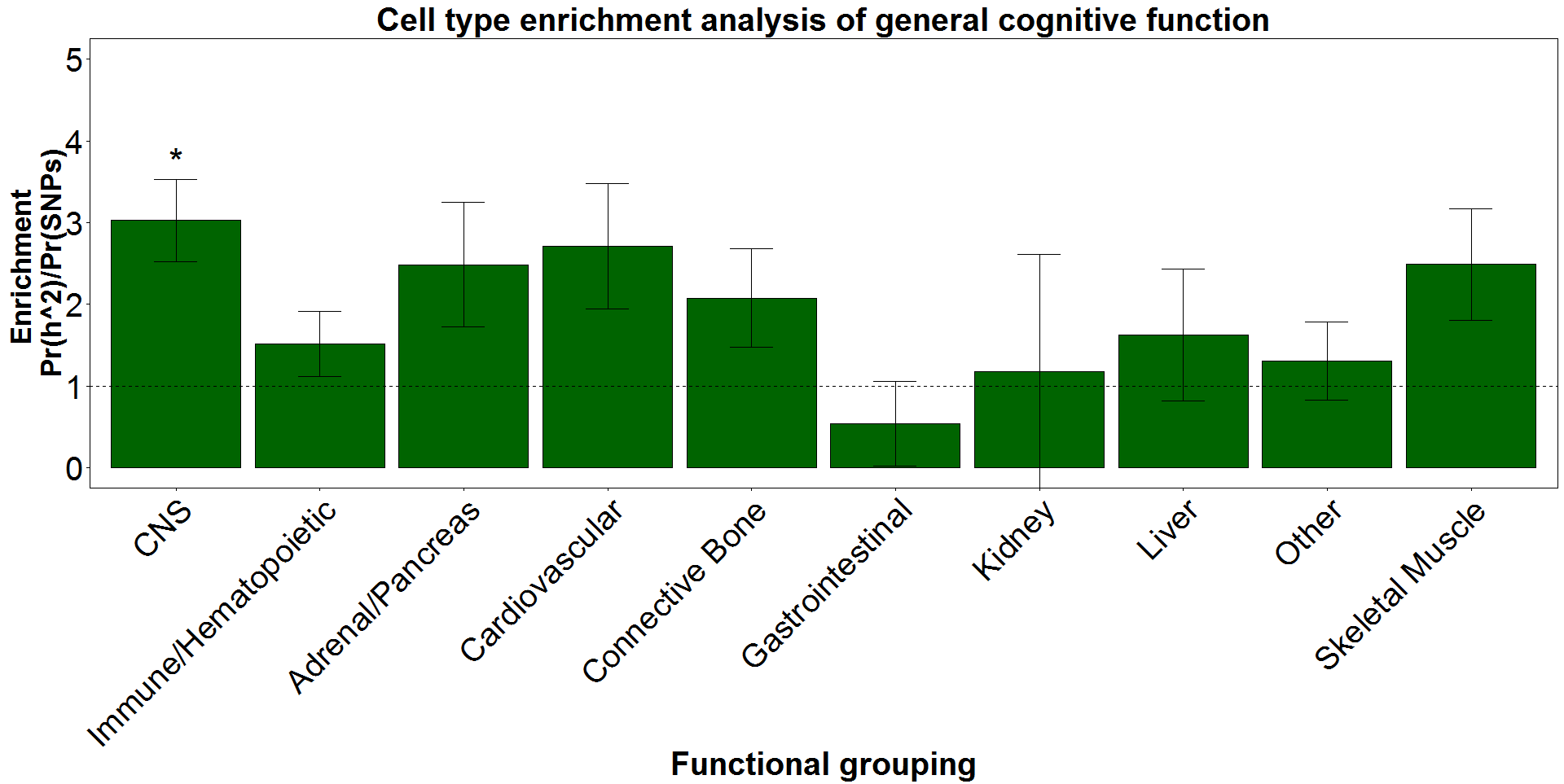


Supplementary Figure 2. Enrichment analysis for general cognitive function using the 10 cell-type specific categories in the baseline model. The enrichment statistic is the proportion of heritability found in each functional group divided by the proportion of SNPS in each group (Pr(h^2^)/Pr(SNPs)). The dashed line indicates no enrichment found when Pr(h^2^)/Pr(SNPs) = 1. Statistical significance is indicated by asterisk. FDR correction indicates significance at 6.37x10^-5^.

Supplementary Figure 3. Enrichment analysis for verbal-numerical reasoning using the 52 functional categories in the baseline model. The enrichment statistic is the proportion of heritability found in each functional group divided by the proportion of SNPS in each group (Pr(h^2^)/Pr(SNPs)). The dashed line indicates no enrichment found when Pr(h^2^)/Pr(SNPs) = 1. Statistical significance is indicated by asterisk. FDR correction indicates significance at 0.004.

Supplementary Figure 4. Enrichment analysis for verbal-numerical reasoning using the 10 cell-type specific categories in the baseline model. The enrichment statistic is the proportion of heritability found in each functional group divided by the proportion of SNPS in each group (Pr(h^2^)/Pr(SNPs)). The dashed line indicates no enrichment found when Pr(h^2^)/Pr(SNPs) = 1. Statistical significance is indicated by asterisk. FDR correction indicates significance at 2.4x10^-5^.

| Functional annotations | Reference |
| --- | --- |
|  |  |
| Coding | Kent, W.J. *et al*. ([1](#_ENREF_1))  Gusev, A. *et al*. ([2](#_ENREF_2)) |
| Conserved | Lindblad-Toh, K. *et al*. ([3](#_ENREF_3))  Ward, L.D. & Kellis, M. ([4](#_ENREF_4)) |
| CTCF | Hoffman *et al. (*[*5*](#_ENREF_5)*)* |
| Digital genomic footprint (DGF) ENCODE | ENCODE ([6](#_ENREF_6))  Gusev, A. *et al*. ([2](#_ENREF_2)) |
| DNase hypersensitivity sites (DHS) | Trynka, G. *et al*. ([7](#_ENREF_7)) |
| FANTOM 5 | Andersson et al. ([8](#_ENREF_8)) |
| Enhancer | Hoffman *et al. (*[*5*](#_ENREF_5)*)* |
| Fetal DNase hypersensitivity sites (DHS) | Trynka, G. *et al*. ([7](#_ENREF_7)) |
| H3K27ac Hnisz | Hnisz, D. *et al*. ([9](#_ENREF_9)) |
| H3K27ac PGC2 | Schizophrenia Working Group of the Psychiatric Genomics Consortium. ([10](#_ENREF_10)) |
| H3K4me1 | Trynka, G. *et al*. ([7](#_ENREF_7)) |
| H3K4me3 | Trynka, G. *et al*. ([7](#_ENREF_7)) |
| H3K9ac | Trynka, G. *et al*. ([7](#_ENREF_7)) |
| Intron | Kent, W.J. *et al*. ([1](#_ENREF_1))  Gusev, A. *et al*. ([2](#_ENREF_2)) |
| Promoter Flanking | Hoffman *et al. (*[*5*](#_ENREF_5)*)* |
| Promoter | Kent, W.J. *et al*. ([1](#_ENREF_1))  Gusev, A. *et al*. ([2](#_ENREF_2)) |
| Repressed | Hoffman *et al. (*[*5*](#_ENREF_5)*)* |
| Super Enhancer | Hnisz, D. *et al*. ([9](#_ENREF_9)) |
| Transcription factor binding site (TFBS) | ENCODE ([6](#_ENREF_6))  Gusev, A. *et al*. ([2](#_ENREF_2)) |
| Transcribed | Hoffman *et al. (*[*5*](#_ENREF_5)*)* |
| Transcriptional start sites (TSS) | Hoffman *et al. (*[*5*](#_ENREF_5)*)* |
| 3-prime UTR | Kent, W.J. *et al*. ([1](#_ENREF_1))  Gusev, A. *et al*. ([2](#_ENREF_2)) |
| 5-prime UTR | Kent, W.J. *et al*. ([1](#_ENREF_1))  Gusev, A. *et al*. ([2](#_ENREF_2)) |
| Weak Enhancer | Hoffman *et al. (*[*5*](#_ENREF_5)*)* |

Supplementary Table 1. Shows the functional annotations and provides a reference detailing how they were curated. These sets are the same as used by Finucane et al., ([11](#_ENREF_11))

References

1. Kent WJ, Sugnet CW, Furey TS, Roskin KM, Pringle TH, Zahler AM, et al. (2002): The human genome browser at UCSC. *Genome research*. 12:996-1006.

2. Gusev A, Lee SH, Trynka G, Finucane H, Vilhjálmsson BJ, Xu H, et al. (2014): Partitioning heritability of regulatory and cell-type-specific variants across 11 common diseases. *The American Journal of Human Genetics*. 95:535-552.

3. Lindblad-Toh K, Garber M, Zuk O, Lin MF, Parker BJ, Washietl S, et al. (2011): A high-resolution map of human evolutionary constraint using 29 mammals. *Nature*. 478:476-482.

4. Ward LD, Kellis M (2012): Evidence of abundant purifying selection in humans for recently acquired regulatory functions. *Science*. 337:1675-1678.

5. Hoffman MM, Ernst J, Wilder SP, Kundaje A, Harris RS, Libbrecht M, et al. (2012): Integrative annotation of chromatin elements from ENCODE data. *Nucleic acids research*.1-15.

6. ENCODE Project Consortium. (2012): An integrated encyclopedia of DNA elements in the human genome. *Nature*. 489:57-74.

7. Trynka G, Sandor C, Han B, Xu H, Stranger BE, Liu XS, et al. (2013): Chromatin marks identify critical cell types for fine mapping complex trait variants. *Nature genetics*. 45:124-130.

8. Andersson R, Gebhard C, Miguel-Escalada I, Hoof I, Bornholdt J, Boyd M, et al. (2014): An atlas of active enhancers across human cell types and tissues. *Nature*. 507:455-461.

9. Hnisz D, Abraham BJ, Lee TI, Lau A, Saint-André V, Sigova AA, et al. (2013): Super-enhancers in the control of cell identity and disease. *Cell*. 155:934-947.

10. Schizophrenia Working Group of the Psychiatric Genomics Consortium (2014): Biological insights from 108 schizophrenia-associated genetic loci. *Nature*. 511:421-427.

11. Finucane HK, Bulik-Sullivan B, Gusev A, Trynka G, Reshef Y, Loh PR, et al. (2015): Partitioning heritability by functional annotation using genome-wide association summary statistics. *Nature genetics*. 47:1228-1235.
