# Supplementary material for: Molecular genetic aetiology of general cognitive function is enriched in evolutionarily conserved regions
Source: Transl Psychiatry. 2016 Dec 13;6(12):e980–. doi: 10.1038/tp.2016.246 (PMC5290340; doi:10.1038/tp.2016.246)
Supplement: Supplementary Tables 2 and 3 [file tp2016246x2.docx]

| Supplementary Table 2. Showing the full output of the partitioned heritability analysis conducted on the general cognitive function data set. Prop. SNPs refers to the proportion of SNPs from the data set that were a part of the corresponding functional annotation. Prop. h2 pertains to the proportion of the heritability accounted for by the functional annotation. | | | | | | |
| --- | --- | --- | --- | --- | --- | --- |
| Category | Prop. SNPs | Prop. h2 | Prop. h2 std error | Enrichment | Enrichment std error | Enrichment p |
| Central Nervous System | 0.149 | 0.450 | 0.075 | 3.025 | 0.506 | 6.37 × 10^−5^ |
| Immune/Hematopoietic | 0.233 | 0.354 | 0.094 | 1.518 | 0.402 | 0.197 |
| Adrenal/Pancreas | 0.094 | 0.233 | 0.071 | 2.486 | 0.759 | 0.050 |
| Cardiovascular | 0.111 | 0.301 | 0.085 | 2.713 | 0.767 | 0.025 |
| Connective Bone | 0.115 | 0.239 | 0.069 | 2.075 | 0.602 | 0.074 |
| Gastrointestinal | 0.168 | 0.090 | 0.087 | 0.538 | 0.517 | 0.372 |
| Kidney | 0.043 | 0.050 | 0.061 | 1.178 | 1.438 | 0.901 |
| Liver | 0.072 | 0.118 | 0.058 | 1.630 | 0.807 | 0.435 |
| Other | 0.203 | 0.264 | 0.097 | 1.304 | 0.478 | 0.525 |
| Skeletal Muscle | 0.104 | 0.259 | 0.071 | 2.491 | 0.683 | 0.029 |
| Coding | 0.015 | 0.146 | 0.065 | 9.962 | 4.441 | 0.044 |
| Coding 500 | 0.065 | 0.217 | 0.066 | 3.365 | 1.028 | 0.021 |
| Conserved | 0.026 | 0.492 | 0.102 | 18.874 | 3.911 | 4.88 × 10^−6^ |
| Conserved 500 | 0.333 | 0.847 | 0.100 | 2.548 | 0.300 | 2.38 × 10^−7^ |
| CTCF | 0.024 | -0.028 | 0.086 | -1.166 | 3.617 | 0.549 |
| CTCF 500 | 0.071 | -0.008 | 0.097 | -0.113 | 1.358 | 0.413 |
| DGF ENCODE | 0.138 | 0.226 | 0.200 | 1.645 | 1.451 | 0.657 |
| DGF ENCODE 500 | 0.542 | 0.410 | 0.154 | 0.757 | 0.284 | 0.393 |
| DHS peaks | 0.112 | 0.336 | 0.208 | 3.006 | 1.865 | 0.282 |
| DHS | 0.168 | 0.586 | 0.219 | 3.490 | 1.305 | 0.056 |
| DHS 500 | 0.499 | 1.022 | 0.164 | 2.049 | 0.328 | 0.001 |
| FANTOM 5 | 0.004 | -0.068 | 0.041 | -15.724 | 9.562 | 0.080 |
| FANTOM 5 500 | 0.019 | -0.031 | 0.051 | -1.631 | 2.669 | 0.324 |
| Enhancer | 0.063 | 0.057 | 0.107 | 0.907 | 1.698 | 0.956 |
| Enhancer 500 | 0.154 | 0.504 | 0.117 | 3.272 | 0.760 | 0.003 |
| Fetal DHS | 0.085 | 0.417 | 0.169 | 4.921 | 1.994 | 0.049 |
| Fetal DHS 500 | 0.285 | 0.578 | 0.140 | 2.026 | 0.493 | 0.037 |
| H3K27ac Hnisz | 0.391 | 0.522 | 0.072 | 1.334 | 0.183 | 0.068 |
| H3K27ac Hnisz 500 | 0.423 | 0.581 | 0.085 | 1.376 | 0.201 | 0.061 |
| H3K27ac PGC2 | 0.269 | 0.333 | 0.120 | 1.237 | 0.445 | 0.594 |
| H3K27ac PGC2 500 | 0.336 | 0.403 | 0.112 | 1.200 | 0.332 | 0.547 |
| H3K4me1 peaks | 0.171 | 0.532 | 0.158 | 3.108 | 0.923 | 0.022 |
| H3K4me1 | 0.427 | 0.739 | 0.147 | 1.732 | 0.345 | 0.034 |
| H3K4me1 500 | 0.609 | 0.875 | 0.093 | 1.436 | 0.152 | 0.004 |
| H3K4me3 peaks | 0.042 | 0.076 | 0.117 | 1.807 | 2.807 | 0.774 |
| H3K4me3 | 0.133 | 0.393 | 0.107 | 2.948 | 0.804 | 0.015 |
| H3K4me3 500 | 0.255 | 0.209 | 0.105 | 0.818 | 0.409 | 0.657 |
| H3K9ac peaks | 0.039 | 0.158 | 0.101 | 4.068 | 2.610 | 0.240 |
| H3K9ac | 0.126 | 0.464 | 0.127 | 3.676 | 1.010 | 0.008 |
| H3K9ac 500 | 0.231 | 0.317 | 0.104 | 1.375 | 0.453 | 0.408 |
| Intron | 0.387 | 0.427 | 0.060 | 1.102 | 0.154 | 0.511 |
| Intron 500 | 0.397 | 0.557 | 0.047 | 1.402 | 0.119 | 0.001 |
| Promoter Flanking | 0.008 | 0.018 | 0.059 | 2.120 | 6.990 | 0.873 |
| Promoter Flanking 500 | 0.033 | 0.080 | 0.066 | 2.399 | 1.958 | 0.475 |
| Promoter | 0.031 | 0.099 | 0.062 | 3.166 | 1.991 | 0.277 |
| Promoter 500 | 0.039 | 0.152 | 0.052 | 3.942 | 1.344 | 0.029 |
| Repressed | 0.461 | 0.534 | 0.149 | 1.158 | 0.323 | 0.624 |
| Repressed 500 | 0.719 | 0.443 | 0.065 | 0.616 | 0.090 | 2.10 × 10^−5^ |
| SuperEnhancer | 0.168 | 0.276 | 0.042 | 1.642 | 0.248 | 0.010 |
| SuperEnhancer 500 | 0.172 | 0.241 | 0.040 | 1.407 | 0.232 | 0.080 |
| TFBS | 0.132 | 0.402 | 0.185 | 3.037 | 1.394 | 0.144 |
| TFBS 500 | 0.343 | 0.441 | 0.164 | 1.283 | 0.477 | 0.553 |
| Transcribed | 0.345 | 0.148 | 0.146 | 0.430 | 0.424 | 0.178 |
| Transcribed 500 | 0.763 | 0.914 | 0.088 | 1.198 | 0.116 | 0.087 |
| TSS | 0.018 | 0.109 | 0.063 | 5.982 | 3.443 | 0.148 |
| TSS 500 | 0.035 | 0.093 | 0.058 | 2.663 | 1.669 | 0.319 |
| 3-prime UTR | 0.011 | -0.003 | 0.038 | -0.232 | 3.471 | 0.723 |
| 3-prime UTR 500 | 0.027 | 0.074 | 0.042 | 2.762 | 1.542 | 0.253 |
| 5-prime UTR | 0.005 | -0.016 | 0.029 | -2.985 | 5.403 | 0.461 |
| 5-prime UTR 500 | 0.028 | 0.135 | 0.049 | 4.854 | 1.749 | 0.028 |
| Weak Enhancer | 0.021 | -2.80 × 10^−4^ | 0.079 | -0.013 | 3.757 | 0.787 |
| Weak Enhancer 500 | 0.089 | 0.381 | 0.092 | 4.282 | 1.030 | 0.001 |

| Supplementary Table 3. Showing the full output of the partitioned heritability analysis conducted on the verbal-numerical reasoning data set. SNPs refers to the proportion of SNPs from the data set that were a part of the corresponding functional annotation. Prop. h2 pertains to the proportion of the heritability accounted for by the functional annotation. | | | | | | |
| --- | --- | --- | --- | --- | --- | --- |
| Category | Prop. SNPs | Prop. h2 | Prop. h2 std error | Enrichment | Enrichment std error | Enrichment p |
| Central Nervous Sysytem | 0.149 | 0.525 | 0.089 | 3.529 | 0.599 | 2.40 × 10^−5^ |
| Immune/Hematopoietic | 0.233 | 0.259 | 0.100 | 1.109 | 0.427 | 0.799 |
| Adrenal/Pancreas | 0.094 | 0.271 | 0.083 | 2.894 | 0.889 | 0.033 |
| Cardiovascular | 0.111 | 0.316 | 0.098 | 2.844 | 0.880 | 0.036 |
| Connective Bone | 0.115 | 0.095 | 0.075 | 0.823 | 0.649 | 0.786 |
| Gastrointestinal | 0.168 | 0.153 | 0.095 | 0.912 | 0.569 | 0.878 |
| Kidney | 0.043 | 0.022 | 0.054 | 0.518 | 1.266 | 0.704 |
| Liver | 0.072 | 0.120 | 0.061 | 1.668 | 0.845 | 0.429 |
| Other | 0.203 | 0.375 | 0.104 | 1.850 | 0.513 | 0.098 |
| Skeletal Muscle | 0.104 | 0.311 | 0.083 | 2.994 | 0.804 | 0.013 |
| Coding | 0.015 | 0.093 | 0.057 | 6.363 | 3.913 | 0.171 |
| Coding 500 | 0.065 | 0.153 | 0.065 | 2.374 | 1.014 | 0.175 |
| Conserved | 0.026 | 0.412 | 0.115 | 15.798 | 4.422 | 0.001 |
| Conserved 500 | 0.333 | 0.872 | 0.127 | 2.624 | 0.383 | 2.25 × 10^−5^ |
| CTCF | 0.024 | 0.030 | 0.102 | 1.279 | 4.279 | 0.948 |
| CTCF 500 | 0.071 | 0.246 | 0.113 | 3.460 | 1.586 | 0.121 |
| DGF ENCODE | 0.138 | 0.030 | 0.226 | 0.221 | 1.642 | 0.635 |
| DGF ENCODE 500 | 0.542 | 0.306 | 0.167 | 0.565 | 0.308 | 0.157 |
| DHS peaks | 0.112 | 0.187 | 0.218 | 1.673 | 1.954 | 0.730 |
| DHS | 0.168 | 0.227 | 0.238 | 1.353 | 1.416 | 0.803 |
| DHS 500 | 0.499 | 0.554 | 0.177 | 1.110 | 0.355 | 0.757 |
| FANTOM 5 | 0.004 | -0.027 | 0.047 | -6.283 | 10.953 | 0.506 |
| FANTOM 5 500 | 0.019 | 0.046 | 0.054 | 2.416 | 2.821 | 0.616 |
| Enhancer | 0.063 | 0.137 | 0.122 | 2.168 | 1.931 | 0.545 |
| Enhancer 500 | 0.154 | 0.271 | 0.102 | 1.764 | 0.662 | 0.249 |
| Fetal DHS | 0.085 | 0.415 | 0.197 | 4.895 | 2.328 | 0.094 |
| Fetal DHS 500 | 0.285 | 0.465 | 0.165 | 1.633 | 0.580 | 0.275 |
| H3K27ac Hnisz | 0.391 | 0.412 | 0.077 | 1.053 | 0.196 | 0.789 |
| H3K27ac Hnisz 500 | 0.423 | 0.619 | 0.090 | 1.464 | 0.214 | 0.030 |
| H3K27ac PGC2 | 0.269 | 0.231 | 0.137 | 0.855 | 0.508 | 0.776 |
| H3K27ac PGC2 500 | 0.336 | 0.379 | 0.108 | 1.128 | 0.320 | 0.690 |
| H3K4me1 peaks | 0.171 | 0.552 | 0.204 | 3.220 | 1.191 | 0.062 |
| H3K4me1 | 0.427 | 0.781 | 0.178 | 1.831 | 0.418 | 0.047 |
| H3K4me1 500 | 0.609 | 0.871 | 0.091 | 1.429 | 0.149 | 0.004 |
| H3K4me3 peaks | 0.042 | 0.028 | 0.133 | 0.676 | 3.194 | 0.919 |
| H3K4me3 | 0.133 | 0.185 | 0.111 | 1.389 | 0.830 | 0.640 |
| H3K4me3 500 | 0.255 | 0.380 | 0.111 | 1.486 | 0.435 | 0.263 |
| H3K9ac peaks | 0.039 | 0.240 | 0.125 | 6.181 | 3.235 | 0.109 |
| H3K9ac | 0.126 | 0.409 | 0.121 | 3.242 | 0.962 | 0.020 |
| H3K9ac 500 | 0.231 | 0.581 | 0.110 | 2.520 | 0.479 | 0.002 |
| Intron | 0.387 | 0.478 | 0.068 | 1.234 | 0.176 | 0.185 |
| Intron 500 | 0.397 | 0.562 | 0.056 | 1.416 | 0.142 | 0.003 |
| Promoter Flanking | 0.008 | -0.008 | 0.054 | -0.915 | 6.364 | 0.764 |
| Promoter Flanking 500 | 0.033 | 0.015 | 0.070 | 0.447 | 2.100 | 0.792 |
| Promoter | 0.031 | -0.001 | 0.061 | -0.046 | 1.951 | 0.592 |
| Promoter 500 | 0.039 | 0.008 | 0.055 | 0.216 | 1.424 | 0.582 |
| Repressed | 0.461 | 0.444 | 0.178 | 0.963 | 0.385 | 0.923 |
| Repressed 500 | 0.719 | 0.622 | 0.067 | 0.864 | 0.093 | 0.144 |
| SuperEnhancer | 0.168 | 0.258 | 0.044 | 1.532 | 0.260 | 0.041 |
| SuperEnhancer 500 | 0.172 | 0.238 | 0.043 | 1.389 | 0.249 | 0.118 |
| TFBS | 0.132 | -0.076 | 0.234 | -0.577 | 1.764 | 0.371 |
| TFBS 500 | 0.343 | 0.718 | 0.177 | 2.091 | 0.516 | 0.035 |
| Transcribed | 0.345 | 0.447 | 0.140 | 1.294 | 0.406 | 0.469 |
| Transcribed 500 | 0.763 | 0.590 | 0.095 | 0.773 | 0.124 | 0.067 |
| TSS | 0.018 | 0.027 | 0.065 | 1.502 | 3.569 | 0.888 |
| TSS 500 | 0.035 | 0.112 | 0.063 | 3.213 | 1.809 | 0.221 |
| 3-prime UTR | 0.011 | 0.070 | 0.071 | 6.355 | 6.418 | 0.404 |
| 3-prime UTR 500 | 0.027 | 0.120 | 0.055 | 4.448 | 2.031 | 0.090 |
| 5-prime UTR | 0.005 | -0.002 | 0.032 | -0.293 | 5.919 | 0.827 |
| 5-prime UTR 500 | 0.028 | 0.028 | 0.050 | 0.997 | 1.797 | 0.999 |
| Weak Enhancer | 0.021 | -0.003 | 0.088 | -0.155 | 4.158 | 0.781 |
| Weak Enhancer 500 | 0.089 | 0.152 | 0.089 | 1.708 | 1.001 | 0.479 |
